# OligoMinerApp: a web-server application for the design of genome-scale oligonucleotide *in situ* hybridization probes through the flexible OligoMiner environment

**DOI:** 10.1093/nar/gkaa251

**Published:** 2020-04-20

**Authors:** Marco Passaro, Martina Martinovic, Valeria Bevilacqua, Elliot A Hershberg, Grazisa Rossetti, Brian J Beliveau, Raoul J P Bonnal, Massimiliano Pagani

**Affiliations:** Istituto Nazionale Genetica Molecolare ‘Romeo ed Enrica Invernizzi’, 20122 Milano, Italy; Department of Medical Biotechnology and Translational Medicine, Università degli Studi di Milano, 20129 Milano, Italy; FIRC Institute of Molecular Oncology (IFOM), 20139 Milan, Italy; Istituto Nazionale Genetica Molecolare ‘Romeo ed Enrica Invernizzi’, 20122 Milano, Italy; Istituto Nazionale Genetica Molecolare ‘Romeo ed Enrica Invernizzi’, 20122 Milano, Italy; Department of Medical Biotechnology and Translational Medicine, Università degli Studi di Milano, 20129 Milano, Italy; Department of Genome Sciences, University of Washington, Seattle,WA 98195, USA; Istituto Nazionale Genetica Molecolare ‘Romeo ed Enrica Invernizzi’, 20122 Milano, Italy; FIRC Institute of Molecular Oncology (IFOM), 20139 Milan, Italy; Department of Genome Sciences, University of Washington, Seattle,WA 98195, USA; Istituto Nazionale Genetica Molecolare ‘Romeo ed Enrica Invernizzi’, 20122 Milano, Italy; FIRC Institute of Molecular Oncology (IFOM), 20139 Milan, Italy; Istituto Nazionale Genetica Molecolare ‘Romeo ed Enrica Invernizzi’, 20122 Milano, Italy; Department of Medical Biotechnology and Translational Medicine, Università degli Studi di Milano, 20129 Milano, Italy; FIRC Institute of Molecular Oncology (IFOM), 20139 Milan, Italy

## Abstract

Fluorescence *in situ* hybridization (FISH) is a powerful single-cell technique that harnesses nucleic acid base pairing to detect the abundance and positioning of cellular RNA and DNA molecules in fixed samples. Recent technology development has paved the way to the construction of FISH probes entirely from synthetic oligonucleotides (oligos), allowing the optimization of thermodynamic properties together with the opportunity to design probes against any sequenced genome. However, comparatively little progress has been made in the development of computational tools to facilitate the oligos design, and even less has been done to extend their accessibility. OligoMiner is an open-source and modular pipeline written in Python that introduces a novel method of assessing probe specificity that employs supervised machine learning to predict probe binding specificity from genome-scale sequence alignment information. However, its use is restricted to only those people who are confident with command line interfaces because it lacks a Graphical User Interface (GUI), potentially cutting out many researchers from this technology. Here, we present OligoMinerApp (http://oligominerapp.org), a web-based application that aims to extend the OligoMiner framework through the implementation of a smart and easy-to-use GUI and the introduction of new functionalities specially designed to make effective probe mining available to everyone.

## INTRODUCTION

Recent advances in molecular technologies have unleashed the power to explore biological processes at single-cell resolution with unprecedented accuracy (1). The improved efficiency of ultra-high-throughput single-cell sequencing system (2), coupled with new bioinformatics approaches for data processing (3), has abruptly led to a massive increase in genetic profiling of cells.

Notwithstanding, ultra-high-throughput single-cell analysis requires cell dissociation, which results in the loss of spatial information and represents a major shortcoming of single-cell methods. Tissues organization often accounts for cell fate differences and lineage determination impacting on their functional role and expression profile (4,5). The combination of gene expression profiles with spatial coordinates of cells, could be achieved using computational methods or relying on direct quantification of mRNA molecules at single cell resolution (6) through fluorescence *in situ* hybridization (FISH). FISH methods have become the golden standard for *insitu* analysis and they have been greatly improved to detect hundreds of genes in thousands of single-cell at a single-molecule resolution (smFISH) (7,8). Together, the imaging-based single-cell technologies allow the dissection of functional state and grouping of individual cells providing the exact copy number of the molecules of interest in the context of their subcellular localization (9).

Started in 1998 by Robert Singer and colleagues (10), the detection of RNA *in situ* is achieved by using a large number of probes targeting a single molecule, each linked to a single fluorophore at their 3′-termini (11). Thus, probe design represents the first mandatory step for approaching nucleic acid *in-situ* hybridization. Despite the advances in DNA technologies, that now allow reliable and fast synthesis of oligonucleotides with several custom modifications, comparatively little progress has been made in computational tools for accurate FISH probes identification. Many utilities designed to assist oligo-pairs design are available as web-server applications or stand-alone packages, in a freeware or proprietary format (12). They offer an overabundance of services such as melting temperature calculation, dimer prediction and specificity of hybridization estimation (12) but they are largely unfit for the requirements of modern FISH technologies where, from hundreds to thousands of different oligonucleotides, have to work together with high specificity. More recent tools, developed for the probe design of microarray approaches, have then been repurposed in the frame of FISH. OligoArray is a program that designs specific oligonucleotides at the genomic scale using a thermodynamic approach to predict secondary structure (13). Despite its successful employment in the design of effective smFISH probe's sets made available through Oligopaints (14), OligoArray does not include the option to fully control and change the values of parameters as desired by the users. Moreover, Oligopaints itself is conceived as bioinformatically designed complex oligonucleotide probe sets for FISH, not allowing users to probe discovery but only let them browse already generated oligos (even in its recent updates). None of the other free or licensed microarray software, such as Picky (15), OligoDB (16), Ospray (currently not available) (17), OligoDesign (18) and AlelleID (http://premierbiosoft.com/bacterial-identification/index.html), has been tested for smFISH applications.

Since 2010, concurrently with the growing emphasis on high-throughput *in*-*situ* technologies, the first releases of software for smFISH probe mining boosted the ongoing experiments. Specifically planned to achieve high sensitivity and specificity of probes through algorithms of growing complexity, new tools such as, mathFISH (19), Oli2Go (20), OligoMiner (21) and LCG Biosearch Technologies Stellaris^®^ (https://www.biosearchtech.com/support/education/stellaris-rna-fish), greatly facilitate researchers in creating the flawless set of probes for their own experiments. Among the new software, mathFISH is not meant for de-novo probes discovery, Oli2Go functionalities are restricted to non-human genomes and Stellaris^®^ offers almost no parameters customization. However, many parameters, such as salt concentration, type of detergent or hybridization temperature, may be crucial for the efficient binding of the probe to its target. These parameters often vary, depending on the experimental needs (followed protocol, biological feature of the sample), therefore it is important to provide users with a fully manageable tool. Moreover, the increasing interest in the identification and quantification of many transcripts in their natural spatial context gave rise to novel smRNA FISH approaches, based on reiterative probe hybridization and stripping procedure (22,23). The possibility to customize probe annealing temperature, as well as the parameters used for the stripping step, would allow a more accurate experimental design. In this framework, OligoMiner can effectively customize *de*-*novo* probe discovery from any genome.

OligoMiner is ‘a rapid and flexible genome-scale design environment for oligo hybridization probes (21)’ written using Biopython (https://www.ncbi.nlm.nih.gov/pubmed/19304878) and conceived as a modular sequence of fully customizable steps that efficiently scan through nucleotide sequences to design reliable smFISH probes. It introduces a supervised machine learning algorithm for assessing probe specificity and it can check for probes specificity on any type of sequenced genome, moreover, the designed probes have been validated with conventional and single-molecule super-resolution microscopy (21). However, lack of any Graphical User Interface (GUI) limits its use to bioinformaticians or researchers who are confident with command line interfaces.

To overcome this limitation and make OligoMiner available to a larger audience, we developed OligoMinerApp, a web-server application that aims to extend the OligoMiner framework through the implementation of a smart and easy-to-use GUI. OligoMinerApp acts as a wrapper of OligoMiner scripts automating all its processes through an underneath novel structure that ensures data reproducibility and framework stability over time. Moreover, it offers new functionalities specially designed to broadly facilitate effective probe mining and data managing to users, on standard and mobile devices.

## MATERIALS AND METHODS

### Application structure

**Figure 1.**
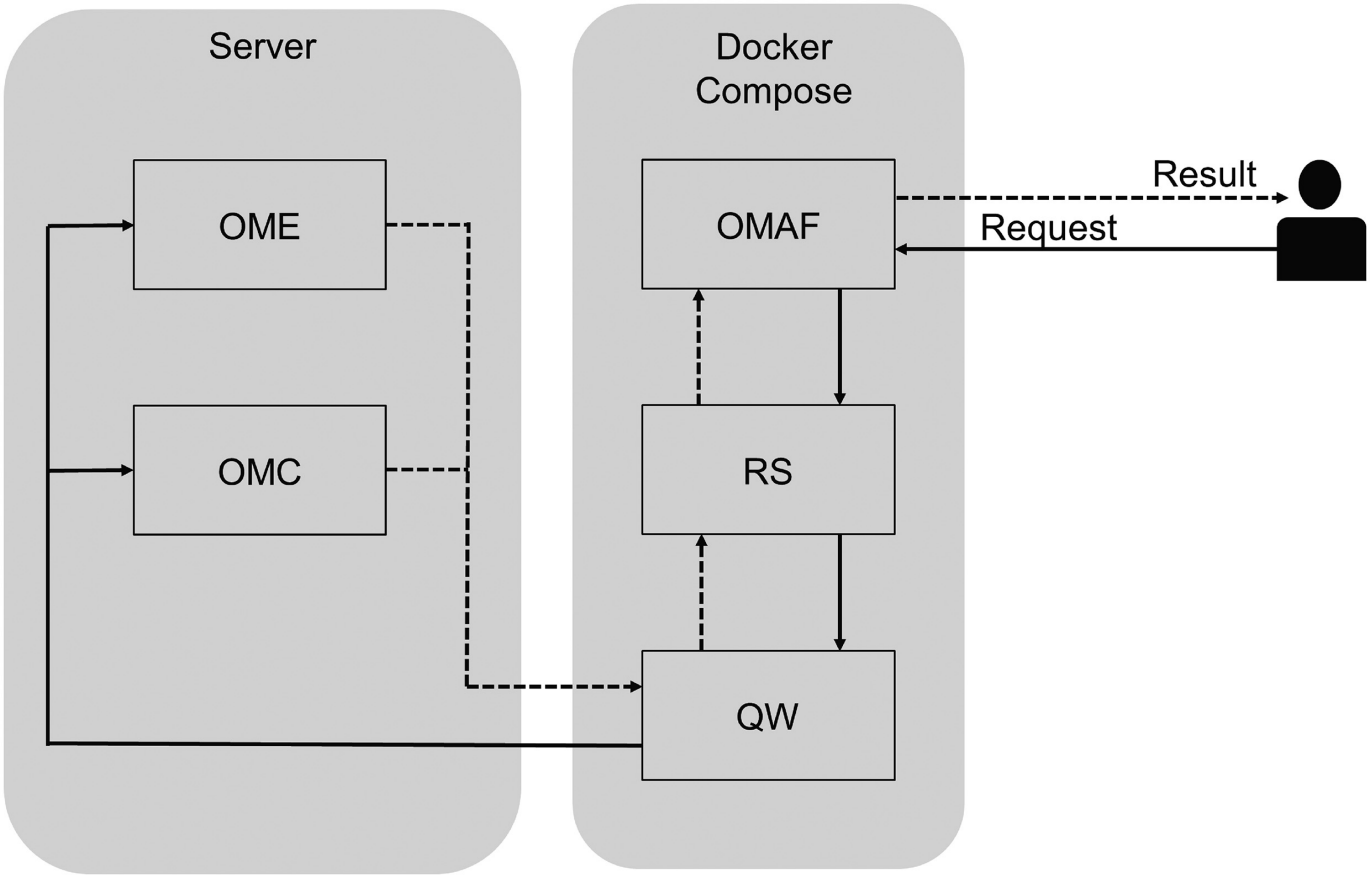
Application structure explaining how the diverse OligoMinerApp modules interact with each other's, from request reception to result generation: ‘OMAF’ manages the requests from users and returns the results of the OligoMiner workflow, ‘OME’ and ‘OMC’ run variations of the OligoMiner scripts consistently with user requests, ‘RS’ and ‘QW’ manage the implemented application queue system avoiding system failure.

OligoMinerApp consists of a back-end service of Python and Bash scripts paired with a front-end interface built in Javascript (JS), Cascading Style Sheets (CSS) and Hyper Text Markup Language (HTML5). The entire application is composed by five independents modules called ‘OligoMinerAppFramework’ (OMAF), ‘OligoMinerEngine’ (OME), ‘OligoMinerCheck’ (OMC), ‘RedisServer’ (RS) and ‘QueueWorker’ (QW), each one encapsulated inside a Docker container under control of the top level Docker–Compose technology (https://www.docker.com/) (Figure 1). Apart from RS Docker Official Image (https://hub.docker.com/_/redis), that is built from Linux Debian distribution, the other Docker images have been created from scratch starting from Linux Ubuntu (Table 1). OMAF represents the main application module and runs Flask (http://flask.pocoo.org/), a micro-framework for Python, BSD licensed, based on Werkzeug WSGI toolkit and Jinja2 template engine. It rules the GUI (front end) by managing HTML5/CSS templates and JS-scripts, assigns the users requests to the correct module through a back-end working-queue and, at last, gives back the results to the front end. The working-queue is created and managed by the concurrent action of OMAF, RS and QW inside the Docker–Compose network using ‘Redis Queue’, a Python library for queueing jobs and processing them in the background on a running Redis server (Figure 1) (Table 1). OME and OMC modules start only upon activation from OMAF on dedicated servers and contain the scripts, tools and dependencies to run variants of the OligoMiner (21) pipeline using Bash and Python. These include Bowtie2 (24), Scikit-learn (25), Jellyfish (26) and NUPACK (27) (Table 1). Each activation involves the request of a OME/OMC module through TCP protocol by setting the default docker daemon to listen on a specific IP address and port instead of the local Unix socket (Figure 1). TCP communication allows OligoMinerApp to remotely control each module configuring it to work with external computing resources if needed. All the software used to run the application are reported in Table 1 with the respective version.

**Table 1.** Technical specifications of images used for running the five OligoMinerApp modules and their main functions: ‘OMAF’, ‘OME’, ‘OMC’, ‘RS’ and ‘QW’, Operative System (OS), requirements (Req.) and function (Fun.)

	OMAF	OME	OMC	RS	QW
OS	Linux Ubuntu 18.04	Linux Ubuntu 18.04	Linux Ubuntu 18.04	Linux Debian: stretch-slim	Linux Ubuntu 18.04
Req	DataTables 1.10.19	Biopython 1.68	Biopython 1.68	Redis-Server 5.0.4	DataTables 1.10.19
	Docker-ce 18.09.04	Bowtie 2.3.4.3	Bowtie 2.3.4.3		Docker-ce 18.09.04
	Flask 1.0.2	Jellyfish 2.2.10	Jellyfish 2.2.10		Flask 1.0.2
	Jinja2 2.10	Miniconda 4.6.11	Miniconda 4.6.11		Jinja2 2.10
	Jquery 3.3.1	Numpy 1.16.2	Numpy 1.16.2		Jquery 3.3.1
	Miniconda 4.6.11	Nupack 3.6.0	Nupack 3.6.0		Miniconda 4.6.11
	Numpy 1.15.3	Pandas 0.24.2	Pandas 0.24.2		Numpy 1.15.3
	Pandas 0.23.4	Python 2.7.15	Python 2.7.15		Pandas 0.23.4
	Pip 10.0.1	Scikit-learn 0.20.3	Scikit-learn 0.20.3		Pip 10.0.1
	Plotly 3.7.1	Scipy 1.2.1	Scipy 1.2.1		Plotly 3.7.1
	Python 3.7.1	Xlrd 1.2.0	Xlrd 1.2.0		Python 3.7.1
	Readline 7.0				Readline 7.0
	Requests 2.19.1				Requests 2.19.1
	Rq 1.0				Rq 1.0
	Xlrd 1.1.0				Xlrd 1.1.0
	Xlswriter 1.1.2				Xlswriter 1.1.2
	Xlutils 2.0.0				Xlutils 2.0.0
	Xlwt1.3.0				Xlwt1.3.0
	Werzeug 0.14.1				Werzeug 0.14.1
Fun	Runs and manages the application back/front-end	Runs single/multi analysis with OligoMiner scripts	Runs probes-check analysis with OligoMiner scripts	Mounts and manages a Redis Server	Creates and manages queue-workers

### Data storage and integrity

The users submitted data undergoes a double integrity verification to avoid the exploitation of bugs caused by code injection vulnerabilities (injection flaws). Data integrity are checked twice: at the front/back end, using HTML5 and Python, to set a limit to size and type. Data are then stored, locally, inside a Microsoft Excel file (.xls) and a text file (.txt) and from it passed to downstream steps. A consistent storage of results is achieved by assign, to every unique user request, a unique numerical code generated by the md5sum algorithm. Md5sum algorithm calculates and verifies 128-bit MD5 hashes that functions as a compact digital fingerprint of each analysis. The code is obtained by digesting an alpha-numerical string representing the junction of data and parameters submitted by the user and it is used to create unique directories for the storage and managing of each analysis data and settings. Exploiting this system, each directory holds variables used as informatics checkpoints, to manage data visibility and privacy inside the GUI of the application searching function. Data storage and handling between the application's modules are entirely managed by Docker Volume technology.

### Dynamic tables, graphs and export functions

Dynamic tables, graphs and exporting options have been obtained by the implementation of JS-scripts inside HTML5 templates of OMAF module. Raw scripts came from DataTables (https://datatables.net/), a plug-in for the jQuery JS library (https://jquery.com/) and Plotly (https://plot.ly/), an open JS library for the creation of interactive charts. Libraries were downloaded from https://cdn.datatables.net, https://cdnjs.cloudflare.com https://code.jquery.com and https://cdn.plot.ly. Graphs were generated inside the back end of the application and then handled through JavaScript Object Notation lightweight data-interchange format.

### Genomes indexing and mapping

All genomes available, were indexed through bowtie2 building option using unmasked reference sequences: ‘Homo_sapiens.GRCh38.dna.primary_assembly.fa.gz’ and ‘Homo_sapiens.GRCh37.dna.primary_assembly.fa’ for *Homosapiens* (ftp://ftp.ensembl.org/pub/release-95/fasta/homo_sapiens/dna/, ftp://ftp.ensembl.org/pub/grch37/release-96/fasta/homo_sapiens/dna/), ‘Mus_musculus.GRCm38.dna.primary_assembly.fa’ for *Musmusculus* (ftp://ftp.ensembl.org/pub/release-96/fasta/mus_musculus/dna/), ‘Rattus_norvegicus.Rnor_6.0.dna.toplevel.fa’ for *Rattusnorvegicus* (ftp://ftp.ensembl.org/pub/release-96/fasta/mus_musculus/dna/), ‘Danio_rerio.GRCz11.dna.primary_assembly.fa’ for *Daniorerio* (ftp://ftp.ensembl.org/pub/release-96/fasta/danio_rerio/dna/), ‘Caenorhabditis_elegans.WBcel235.dna.toplevel.fa’ for *Caenorhabditiselegans* (ftp://ftp.ensembl.org/pub/release-96/fasta/caenorhabditis_elegans/dna/), ‘Drosophila_melanogaster.BDGP6.22.dna.toplevel.fa’ for *Drosophilamelanogaster* (ftp://ftp.ensembl.org/pub/release-96/fasta/drosophila_melanogaster/dna/) and finally ‘Arabidopsis_thaliana.TAIR10.dna.toplevel.fa’ for *Arabidopsisthaliana* (ftp://ftp.ensemblgenomes.org/pub/release-43/plants/fasta/arabidopsis_thaliana/dna/). Bowtie2 mapping of probes is performed by suppressing SAM file header lines (–no-hd) and allowing the searching of up to 100 valid alignments for each probe (-k 100) with high sensitivity and accuracy (–very-sensitive). Jellyfish files (.jf), needed for counting k-mers inside reference DNA sequences, were obtained using an hash of 3300 million elements (-s 3300), nine different k-mers sizes for each reference genome (-m ‘k-mer length’), a counter size of 1 byte to reduce output file size (–out-counter-len 1) and without reporting k-mers that only occur once (-L 2). All these commands were set up following the suggestions provided by the authors of OligoMiner (https://github.com/brianbeliveau/OligoMiner).

### Benchmarking

To monitor the time OligoMinerApp needs to perform probes mining and filtering, we implemented the GNU ‘time’ executable functions in the main code. Starting from a Genome Reference Consortium Human Build 38 (GRCh38) sequence of 170 kbp we generated different random sets of fasta sequences and probes. Seven fasta sequences, respectively 300 bp, 500 bp, 800 bp, 1 kbp, 10 kbp, 50 kbp and 100 kbp long, were tested 10 times each, through the ‘Input—Single’ function of the application. Seven probe's set, counting respectively 10, 40, 80, 100, 1000, 1500 and 3000 probes (each of 30 bp), were tested 10 times each, through the ‘Input—Probes Check’ function of the application. The data from each test were elaborated through ‘jupyter notebook’ using ‘statistics’, ‘matplotlib’, ‘numpy’ and ‘pingouin’ python packages.

## RESULTS

OligoMiner is written in Python using Biopython and is assembled in five independent Python scripts, each one performing a functional step in the probe discovery workflow (21). The first step searches for DNA oligonucleotides (oligos) relying on the input sequence submitted by the user. Bowtie2 then aligns the discovered probes on the desired reference genome, the following step then uses a Linear Discriminant Analysis (LDA) model to screen for oligos that are likely to have off-target affinity. The remaining sequences are finally screened against high abundance k-mers and checked for secondary structures. All these steps are runnable on Linux or Mac OS X systems through the Bash Shell command line.

### Graphical user interface

The majority of scientists are not used to work through scripting and the most adopted Operative System (OS) by institutions is Microsoft Windows. Therefore, we developed a GUI available from browsers, like Firefox or Chrome, thus bypassing OS restrictions and expanding the possibilities for OligoMiner utilization. The visual environment is composed by a dynamic menu beside the main field, where the user is called to input sequences and parameters by writing, or copy and paste, them inside the preconfigured boxes or by directly uploading a fasta file from his/her own computer. In case of errors, miss-clicking or data inconsistency, the system prevents the pipeline from launching by reporting error messages in the corresponding failed submission-boxes. The implementation of a GUI interface created a smart and easy-to-use environment for all users, offering a strategic positioning of HTML button links pointing to helping modal windows, default values for each settable parameter and small help text that appear by passing the mouse pointer over different areas of the screen.

### Input/output, storage and privacy handling

Because OligoMiner is sensitive to the structure and character composition of the input files, to avoid bugs during analysis, we developed a series of Python scripts that sanitize input sequences, however, letting the user to maintain the desired sequences’ names. During input submission, we added the possibility to define on which DNA strand (sense or antisense) define the resulting probes, making OligoMinerApp effective also in managing antisense/non-coding RNA or DNA mobile genetic elements. Once the user starts the discovery process, the submitted files, parameters, results and other intermediates generated by the pipeline, are stored inside a unique directory, ensuring storage consistency. On the other hand, for each analysis a unique code is given, allowing the user to recover pipeline's results and settings through the implemented searching function, which, at the same time, avoid to engage the server resources in repeat identical requests. To overcome privacy issues, the application could either publicly share the stored analyses or grant the access to them through the implemented sharing function accessible by the menu. Independent of the user's choice, OligoMinerApp will store data for 90 days before deleting them from the server. We further improved the way OligoMiner generates output by adding a wide range of exporting options together with the possibility to dynamically browse results inside the GUI, without necessarily downloading them. We provided pre-formatted outputs for Adobe and Microsoft Office formats together with the chance to directly print them on paper or on clipboard. Moreover, each result's page is correlated with graphs, comparing the melting and the stripping temperatures distributions and values.

### Processes automation

OligoMinerApp enables three types of analysis. Each one is developed by automating the processing of OligoMiner stand-alone modules, delegating to the application the managing of intermediates, temporary files and the sequencing of scripts, so that the user will submit input and parameters only once. The ‘Input-Single’ is the main function of OligoMinerApp, representing the standard workflow for probes discovery inside the framework of OligoMiner. With ‘Input–Multi’, we implemented the simultaneous submission of up to 40 sequences to be analyzed together under the same experimental conditions, channeling all final sets of probes into a single sorted output that is encapsulated and automatically managed inside a single working process. In both cases OligoMinerApp accepts fasta sequences up to 100 Kb long. The faculty to evaluate probes coming from other sources (e.g. other software or available probes collections), is provided by ‘Input—Probes Check’ functionality. It includes bowtie2 alignment, LDA screening, k-mers off-target evaluation and secondary structure forecast. The function accepts up to 3000 probes in fasta and automatically formats data to be inserted into the pipeline variation.

### Queue management

OligoMiner system requirements vary during processing according to the running module, with a bottle-neck in correspondence with Jellyfish execution. Indeed, Jellyfish analysis uses up to 50% of the available Random Access Memory (RAM) on our Linux server (16 GB of RAM), resulting in an estimated memory usage of 6–8 GB. Moreover, depending on the input data, it represents the longest step to perform inside the OligoMiner pipeline. Given these premises, running three parallel analyses gives rise to a system's crash. In the perspective of morphing OligoMiner into a web application potentially habitable by dozens of users at the same time, we implemented a queue management system able to process all the simultaneously requests one by one, avoiding system issues and ensuring application effectiveness over time.

### Data reproducibility

Bioinformatics tools resemble a double-edged sword. If on the one hand they have greatly improved the effectiveness and scope of data processing, on the other they are extremely prone to data reproducibility issues. Indeed, the plethora of updates that a bioinformatics tool has to put up combined with the diversity of informatics environments where pipelines are run, cannot assure data firmness. To overcome this issue, OligoMinerApp pipelines run on isolated OS not subjected to external perturbations and fixed over time, allowing tools to maintain the same behavior hence safeguarding consistency of results. To this aim we used the containerization technology, through which all the application's components are encapsulated and thus maintained unchanged over time independently of the hosting workstation or OS.

### Benchmarking

**Figure 2.**
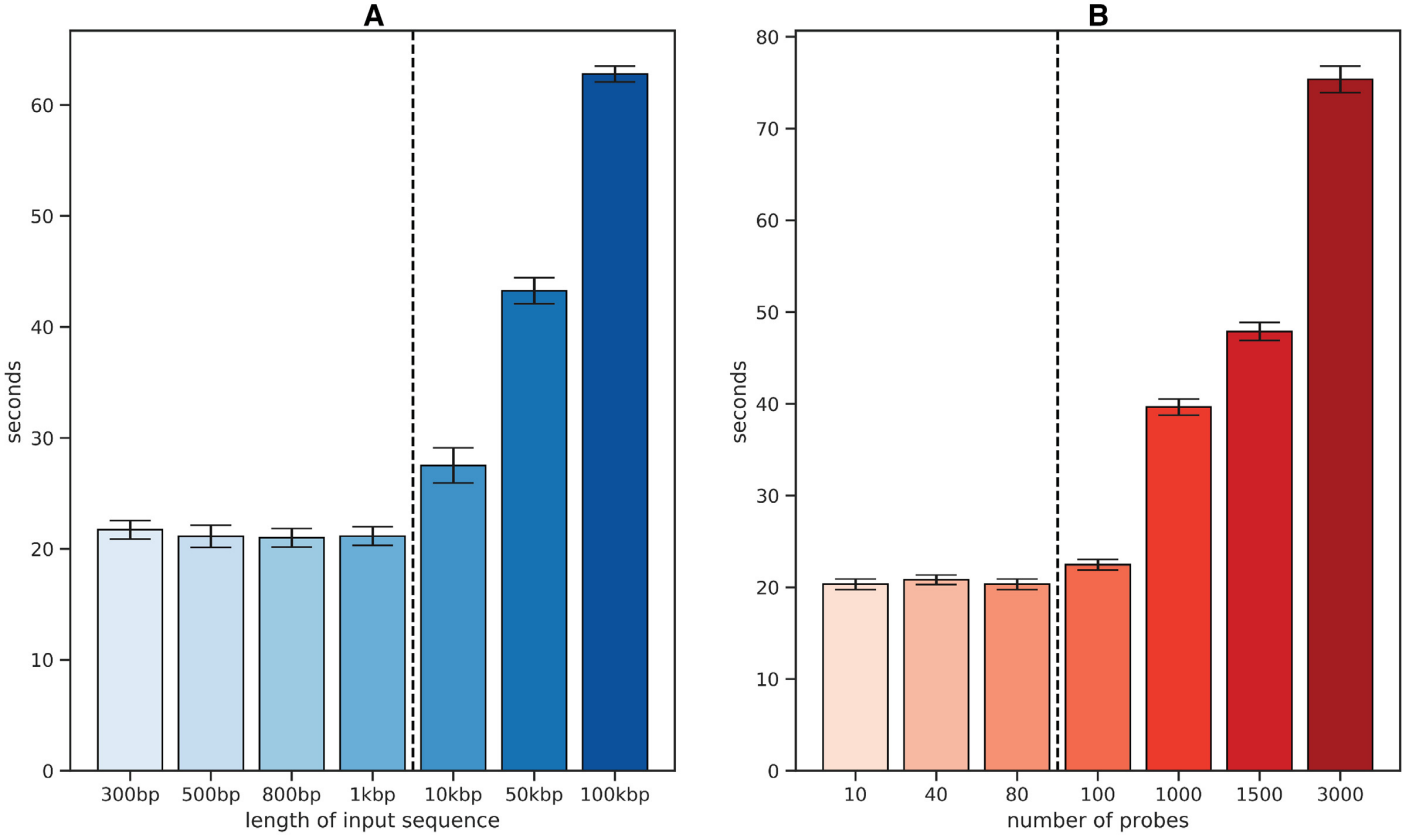
Bar plots of the time that OligoMinerApp needs to analyze data. Each bar shows the mean value and standard deviation of 10 independent tests for a specific type of input; the mean values to the left of the dashed vertical line are not statistically different. (**A**) Mean time, expressed in seconds, that OligoMinerApp needs to mine probes on input fasta sequences of different lengths. (**B**) Mean time, expressed in seconds, that OligoMinerApp needs to filter set of probes of various size.

OligoMinerApp typically can process sequences of 1, 10, 50 and 100 kbp in about 21, 27, 43 and 62 s, respectively (Figure 2A). However, when testing for input sequences smaller than 1 kbp, the time needed to complete the processes does not statistically differ (ANOVA *P*-value = 0.27) (Figure 2A). Basing on these data we evaluated that each mining process roughly takes a fixed time of 20 s plus 0.5 s for each kilobase of the input sequence. It is possible to slightly reduce the processing time of about 10–30% by using the ‘Input–Multi’ function when possible (data not shown). Likewise, OligoMinerApp takes about 22, 39, 47 and 75 s to process probe's set composed by 100, 1000, 1500 and 3000 probes, through the ‘Input—Probes Check’ function (Figure 2B). Also in this case, when testing for smaller probe's set (lower than 80 probes) the time needed to complete the processes does not statistically differ (ANOVA *P*-value = 0.08) (Figure 2B) suggesting a fixed processing time of roughly 20 s plus 0.2 s every 10 probes.

## DISCUSSION

Overall, it is clear that the recent technological progresses in life sciences have the power to bring out completely new perspectives about biological mechanisms, deepening our knowledge and fuelling the advancements of scientific research. However, they often remain accessible only through command line interfaces confirming the negative trend that associates, to a growing computing power, a decreasing user's accessibility. This is particularly true when talking about molecular technologies related to single cells approaches involving, not only data analysis, but also experimental design. SmFISH technologies, indeed, require a carefully designed and functional set of probes in order to gain readable pictures up to 200 nm resolution. This level of specialization is achieved by the employment of bioinformatics tools that combine various approaches to probes mining, but rarely implement a useful interface for non-bioinformatics researchers.

According to this trend, OligoMiner represents one of the most powerful and customizable tools for probe design but lacks any type of interface. Trying to go against the tide and driven by the idea that accessibility is the key feature to promote technology, we morph OligoMiner package into a standalone web application named OligoMinerApp. Available on every HTML5-compatible browser, the application offers a smart and easy-to-use GUI able to collect input sequences and parameters from users, covering all the OligoMiner customizations, thus allowing a mining of probes aware of experimental conditions. Inside OligoMinerApp, the scripts that constitute a cycle of mining run automatically for each user request and are able to manage up to 40 sequences together in the same process. This results in a remarkable saving of time compared to the necessity to run each OligoMiner step individually using the command line. Moreover, we tried to meet user's needs by offering different exporting formats for outputs, a dynamic exploration of results directly inside the application, an interactive visualization and comparison of melting/stripping temperatures, and a remote storage service aware of privacy needs of institutions. At last, a queue managing system allows multiple analyses to be launched at the same time without reaching hardware limitations of server and the implemented container technology assures data reproducibility over time. All together, these features support our idea of harnessing power through accessibility configuring OligoMinerApp as a boosted graphical implementation of OligoMiner package.

We developed OligoMinerApp with the idea that technology should be double-sided by performing complex operations while being easily serviceable by users. In this context we provide a new tool that mounts the power of OligoMiner package inside a smart interface aiming to extend its framework thus allowing more people to generate an effective set of probes for smFISH approaches. Moreover, we do not think of OligoMinerApp as a static tool, on the contrary we plan to continuously update and upgrade it following the needs of users and advances in technology. OligoMinerApp is reachable at the following address: http://oligominerapp.org.

## DATA AVAILABILITY

OligoMinerApp (http://oligominerapp.org)

OligoArray (http://nebc.nox.ac.uk/bioinformatics/docs/OligoArray.html)

Oligopaints (https://oligopaints.hms.harvard.edu/probe-mining-oligoarray)

Picky (https://www.complexcomputation.org/download/Picky/)

OligoDB (http://berry.engin.umich.edu/oligoarray2)

Ospray (http://osprey.ucalgary.ca)

OligoDesign (http://lnatools.com/)

AlleleID (http://premierbiosoft.com/bacterial-identification/index.html)

MathFISH (mathfish.cee.wisc.edu)

Oli2Go (http://oli2go.ait.ac.at/)

OligoMiner (https://github.com/beliveau-lab/OligoMiner)

Stellaris (https://www.biosearchtech.com/support/education/stellaris-rna-fish)

Docker-Technology (https://www.docker.com/)

Flask (http://flask.pocoo.org/)

Bowtie2 (http://bowtie-bio.sourceforge.net/bowtie2/index.shtml)

Scikit-learn (https://scikit-learn.org/stable/)

Jellyfish (https://www.cbcb.umd.edu/software/jellyfish/)

NUPACK (http://www.nupack.org/)

DataTables (https://datatables.net/)

jQuery JS library (https://jquery.com/)

Plotly (https://plot.ly/)

*H. sapiens* genomes (ftp://ftp.ensembl.org/pub/release-95/fasta/homo_sapiens/dna/, ftp://ftp.ensembl.org/pub/grch37/release-96/fasta/homo_sapiens/dna/)

*M. musculus* genome (ftp://ftp.ensembl.org/pub/release-96/fasta/mus_musculus/dna/)

*R*. *norvegicu* genome (ftp://ftp.ensembl.org/pub/release-96/fasta/mus_musculus/dna/)

*D. rerio* genome (ftp://ftp.ensembl.org/pub/release-96/fasta/danio_rerio/dna/)

*C. elegans* genome (ftp://ftp.ensembl.org/pub/release-96/fasta/caenorhabditis_elegans/dna/)

*D. melanogaster* genome (ftp://ftp.ensembl.org/pub/release-96/fasta/drosophila_melanogaster/dna/)

*A. thaliana genome* (ftp://ftp.ensemblgenomes.org/pub/release43/plants/fasta/arabidopsis_thaliana/dna/)
